# Herpetic Keratitis Preceded by COVID-19 Vaccination

**DOI:** 10.3390/vaccines9121394

**Published:** 2021-11-25

**Authors:** Saiqun Li, Xiuhua Jia, Fei Yu, Qian Wang, Tingting Zhang, Jin Yuan

**Affiliations:** 1 State Key Laboratory of Ophthalmology, Zhongshan Ophthalmic Center, Sun Yat-Sen University, Guangzhou 510623, China; xiaolu7886@126.com (F.Y.); wangqian920920@foxmail.com (Q.W.); wangjbnew@163.com (T.Z.); 2 Department of Ophthalmology, The Third Affiliated Hospital of Sun Yat-Sen University, Guangzhou 510630, China; jiaxiuh@mail.sysu.edu.cn

**Keywords:** herpetic keratitis, COVID-19 vaccination, inactivated vaccine

## Abstract

The global Coronavirus Disease 2019 (COVID-19) pandemic has accelerated vaccine development at an unprecedented rate. A large population of people have received COVID-19 vaccines, while the vaccine safety data are limited. Here, we reported two cases of herpetic keratitis that occurred soon after receiving the inactivated COVID-19 vaccines. Case 1 was a 60-year-old woman who underwent penetrating keratoplasty (PKP) one year ago for corneal scarring caused by herpes simplex keratitis (HSK), and case 2 was a 51-year-old man with an unremarkable medical history. Both patients developed herpetic keratitis (HSK and varicella-zoster virus corneal endotheliitis, respectively) soon after receiving the inactivated COVID-19 vaccines (Sinovac). Herpetic keratitis was treated successfully with topical or plus oral antiviral ganciclovir. The short latency time in these two cases suggested that an inactivated COVID-19 vaccine may have a risk of triggering ocular herpes virus reactivation. Both clinicians and patients should be aware of this phenomenon. However, a causal relationship awaits confirmation.

## 1. Introduction

The Coronavirus Disease 2019 (COVID-19) pandemic, caused by severe acute respiratory syndrome coronavirus 2 (SARS-CoV-2), continues to spread worldwide at an accelerated rate, with hundreds of millions of confirmed cases across nearly 200 countries. Vaccination is an effective approach to alleviate the current pandemic [1]. Globally there are twelve COVID-19 vaccine candidates approved for use, and many more are under clinical or pre-clinical development process [2]. Results from large clinical trials have proved the protection effect of vaccines against COVID-19 infection and transmission [3,4]. The global pandemic necessitates vaccine development and approval at an unprecedented speed. The consequence of this is that the data required for specialists to identify vulnerable subpopulations for special considerations or advice is yet relatively limited.

The severe adverse events (SAE) related to COVID-19 vaccines are rare [5]. Most are transient and mild and can resolve on their own. Ocular adverse events present after COVID-19 vaccination have been reported, among which corneal transplant rejection is the most common [6,7]. Others include uveitis and retinal abnormalities [8,9,10]. Here, we reported two cases of herpetic keratitis that occurred soon after COVID-19 immunization, but whether the herpetic keratitis is a real adverse reaction or a coincidental event is not established definitively.

## 2. Case Presentation

**Figure 1:**
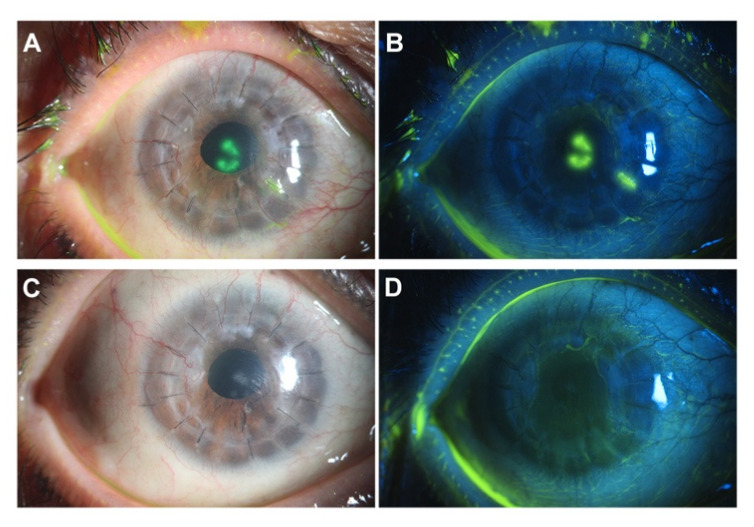
Reactivation of herpes simplex keratitis (HSK) following Coronavirus Disease 2019 (COVID-19) vaccination. (**A**,**B**) Slit-lamp examination one week postvaccination showed a typical herpes simplex viral (HSV) dendritic lesion in the corneal graft center. (**C**,**D**) The keratitis had resolved after a two-week antiviral treatment.

Patient 1 was a 60 year old woman who presented tearing associated with redness, photophobia and worsened vision in the right eye starting from two days after receiving her first dose of inactivated COVID-19 vaccine (Sinovac, Beijing, China). She had no relevant family history. Her medical history was unremarkable with the exception that she had undergone penetrating keratoplasty (PKP) in the same eye one year ago for corneal scarring caused by herpes simplex keratitis (HSK). Her HSK had been quiescent for years. The postoperative medical regimen included topical steroids, tacrolimus and artificial tears. She had ceased to use either topical or systemic antiviral therapy after one month postoperatively. The transplanted cornea maintained transparency with no evidence of recurrence until recently. At presentation, this time, the slit-lamp examination showed a typical herpes simplex viral (HSV) dendritic lesion in the corneal graft center (Figure 1A,B). HSV-1 DNA was isolated from lesion scrapings. The lesion scrapings were negative for other herpetic viruses, including varicella-zoster virus (VZV), cytomegalovirus (CMV) and Epstein–Barr virus (EBV). Herein, a diagnosis of recurrent herpes simplex epithelial keratitis was made. The patient was additionally treated with topical ganciclovir but discontinued the use of topical steroids. Over a period of two weeks, the keratitis had resolved (Figure 1C,D). During the treatment, the patient received her second dose of COVID-19 vaccination (Sinopharm, Beijing, China) without an exacerbation of the existing HSK.

**Figure 2:**
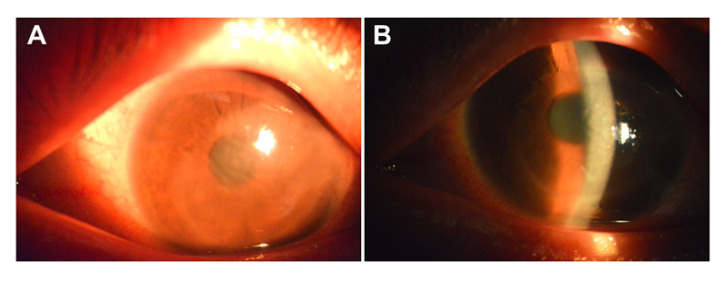
Varicella-zoster virus (VZV) corneal endothelitis following Coronavirus Disease 2019 (COVID-19) vaccination. (**A**) At presentation, the slit-lamp examination revealed diffused corneal edema associated with underlying Descemet’s fold and keratic precipitates. (**B**) The corneal edema was significantly reduced after a one-week topical and oral antiviral treatment.

Patient 2 was a 51-year-old man who presented with redness and blurry vision in his left eye a couple of days after his second dose of COVID-19 vaccination (Sinovac, China). He was well otherwise, and there was no relevant family history. The ocular and medical history were also unremarkable. No abnormalities were reported after his first dose of COVID-19 vaccination (Sinopharm, China). He did not respond well to more than a one-month treatment of topical steroids. At presentation to our ophthalmic clinic, the slit-lamp examination revealed diffused corneal edema associated with underlying Descemet’s fold, keratic precipitates and anterior chamber inflammation (Figure 2A). Quantitative polymerase chain reaction (PCR) detected significant viral loads of VZV DNA in the aqueous humor sample. Herein, herpetic corneal endotheliitis was diagnosed. His treatment regimen included topical steroids as well as topical and oral ganciclovir. The corneal edema was significantly reduced after a one-week treatment (Figure 2B).

## 3. Discussion

Since December 2020, the global number of laboratory-confirmed COVID-19 cases has exceeded 200 million, causing countless losses of life and massive social and economic disruption. Hence, there is an urgent need to develop a safe and effective vaccine to help bring an end to this pandemic. To date, a number of COVID-19 vaccines have been authorized for use, each with different mechanisms of action [11]. Protein subunit vaccines make up the majority of COVID-19 vaccine candidates in clinical trials, while mRNA vaccines that encode viral proteins have several beneficial features over other types of vaccines in terms of safety, efficacy and production [12]. China’s COVID-19 vaccines currently in use (Sinovac and Sinopharm) are based on an inactivated form of SARS-CoV-2.

While COVID-19 vaccines have been evaluated for safety, their adverse event profile remains to be elucidated in full. As huge vaccination programs are ongoing, specialists are witnessing a variety of mild-to-moderate adverse events that imply a possible causal link to COVID-19 vaccination. Suspect ocular adverse events following COVID-19 vaccination are uncommon. Several cases of corneal allograft rejection following immunization with the COVID-19 mRNA vaccine BNT162b2 have been reported [6,7,13]. Further, a case of acute uveitis after the second dose of an inactivated COVID-19 vaccine (Sinopharm) has also been described. [8] Recently, Pichi et al. reported seven cases of potential ocular complaints including retinal abnormalities following COVID-19 vaccination (Sinopharm) [9]. Although an increased number of VZV and HSV dermatitis cases preceded by COVID-19 vaccines has been reported [14], post-COVID-19 vaccination herpetic keratitis is rarely reported in the literature [15]. Before our cases, there was only one report of HSK recurrence after receiving an adenovirus-vectored COVID-19 vaccine (AstraZeneca). According to these few existing reports, ocular adverse event manifestations occurred within two weeks of COVID-19 vaccination and can appear as early as the day after vaccination [6,7,8,9,13,16]. Various types of COVID-19 vaccines such as mRNA vaccine [7], inactivated vaccine [9] and recombinant vector vaccine [16] can all be involved.

It should be kept in mind that ocular adverse events are not exclusive to COVID-19 vaccines and have been reported in many other vaccines, such as influenza, zoster, tetanus and pneumococcal vaccines. Reactions of eyelids and conjunctiva are the most commonly reported complications. Other frequent ocular adverse events include optic neuritis and various patterns of intraocular inflammation [17]. Keratitis is a probable but uncommon vaccine-associated adverse events. Grillo et al. had previously identified 24 cases of keratitis after receiving a live attenuated VZV vaccine [18]. Vaccinations against non-herpes virus, especially influenza vaccinations, have the risk of leading to the reactivation of ocular herpes virus infection like what happened in our cases. For instance, Rothova et al. had reported a case of VZV-related acute retinal necrosis (ARN) following flu H1N1 vaccination [19].

In our two patients, the exact mechanisms that trigger the reactivations of herpes viral infection after an inactivated COVID-19 vaccination remain elusive. Neurotropic HSV and VZV establish a latent infection for the entire life of the host, and their reactivations have been attributed to insufficient cellular immunity [20]. Vaccine-induced immunomodulation (e.g., immunosuppressive effect, decreased alloreactivity) has been previously documented in the literature [21]. Therefore, a temporary decrease of cell-mediated immunity during the early period post vaccination may be involved. Other proposed mechanisms include molecular mimicry and the distraction of the humoral response [16,22].

## 4. Conclusions

To our knowledge, this is the first report of herpetic keratitis with a clear temporal relationship to inactivated COVID-19 vaccination and the first report of VZV corneal endothelitis following any immunization. Ophthalmologists and primary care physicians should be aware of this possible complication if a patient presents ocular pain and a change in vision after COVID-19 immunization. However, given the fact that both COVID-19 vaccination and herpes viral reactivation is frequent, it is very difficult to rule out a fortuitous event. Moreover, even if herpes viral reactivation is a real vaccine-associated adverse event, we do not suggest withholding COVID-19 vaccination because its incidence is extremely low. There is also no sufficient evidence to support the necessity for prophylactic antiviral therapy in people with a history of herpetic keratitis. The question of whether patients with active herpetic keratitis should delay COVID-19 vaccination warrants future investigation.

## Data Availability

The data presented in this study are available upon request from the corresponding authors.
